# Impact of levetiracetam and ethanol on memory, selected neurotransmitter levels, oxidative stress parameters, and essential elements in rats

**DOI:** 10.1007/s43440-024-00659-5

**Published:** 2024-10-01

**Authors:** Ewa Zwierzyńska, Michał Klimczak, Marzenna Nasiadek, Joanna Stragierowicz, Bogusława Pietrzak

**Affiliations:** 1 Department of Pharmacodynamics, Medical University of Lodz, Muszyńskiego 1, Łódź, 90-151 Poland; 2 Department of Toxicology, Medical University of Lodz, Muszyńskiego 1, Łódź, 90-151 Poland

**Keywords:** Ethanol, Levetiracetam, Memory, Neurotransmitter, Oxidative stress, Trace elements

## Abstract

**Background:**

Ethanol disrupts brain activity and memory. There is evidence supporting the beneficial effect of levetiracetam on alcohol consumption. Therefore, the aim of the study was to examine whether levetiracetam has a protective activity against ethanol-induced memory impairment, alterations in selected neurotransmission activities, oxidative stress, and selected essential elements in rats.

**Methods:**

The rats were given levetiracetam (300 mg/kg b.w. *po*) with ethanol for three weeks prior to behavioral tests. Spatial memory was tested using the Morris water maze, while recognition memory was evaluated using the Novel object recognition test. The GABA and glutamate concentration was determined in three rat brain regions (cerebellum, hippocampus, and cerebral cortex). Serum oxidative stress parameters and selected essential elements concentration (Cu, Mn, Zn, Fe, Mg) in the rat brain were analyzed.

**Results:**

Levetiracetam administered with ethanol improved spatial memory, but did not affect abstinence-induced impairment. The drug also decreased ethanol-induced long-term recognition memory impairment. No alterations in glutamate levels were observed. GABA levels were elevated by levetiracetam in the cerebral cortex and by ethanol in the cerebellum. Ethanol increased catalase activity (CAT) and decreased superoxide dismutase activity (SOD) in the serum. Levetiracetam significantly increased the activity of SOD. Alcohol disrupted the levels of trace elements (Mn, Zn, Mg) in the rat brain. Additionally, levetiracetam alone increased Mg, Fe, and Cu concentrations while all animals receiving the drug also had significantly lower concentrations of Zn.

**Conclusions:**

Levetiracetam had differential effects against ethanol-induced impairments. These findings could have important implications for future levetiracetam treatment in patients.

**Supplementary Information:**

The online version contains supplementary material available at 10.1007/s43440-024-00659-5.

## Introduction

Levetiracetam is an antiseizure medication with a broad spectrum of activity. Both preclinical and clinical data indicate that levetiracetam may reduce alcohol consumption [1–3], although some studies report a contrary effect [4, 5]. It is known that alcohol abuse can lead to cognitive impairment and neurodegeneration. Ethanol disrupts hippocampal function and contributes to the deleterious impacts on learning and memory [6]. Our previous pharmaco-EEG study showed reduced hippocampal sensitivity to the impact of ethanol after administration of levetiracetam [7]. In the clinical trial, levetiracetam did not cause working memory and verbal fluency impairment in patients with alcohol dependence compared to topiramate and zonisamide [2].

Although the mechanism of action of levetiracetam is not fully understood, it is known to differ from that of conventional antiseizure medications. It is believed that it is mainly related to binding to the synaptic vesicle 2 A (SV2A) protein [8] and the inhibition of the high-voltage-activated calcium channels [9]. The function of SV2A remains unclear, but the protein seems to be involved in the function of synaptic vesicles such as calcium-dependent exocytosis or transport of vesicle constituents [10]. It has been shown that SV2A is expressed in γ-aminobutyric acid- (GABA)ergic and glutamatergic neuron terminals in the hippocampus and may improve the excitatory/inhibitory balance [11]. SV2A regulates neurotransmitter release, and changes in its expression are linked to alterations in GABAergic and glutamatergic neurotransmissions [12–14]. Levetiracetam may affect GABAergic and glutamatergic neurotransmissions, but the exact mechanism is not yet known. It has been recently shown that levetiracetam suppresses astroglial glutamate release through the inhibition of SV2A expression [15]. This inhibitory effect on glutamate transmission may also be related to P/Q-type calcium channels [16] or glutamate and GABA transporters [17]. Levetiracetam can also reverse the Zn²-induced suppression of GABA-mediated inhibition, resulting in a presynaptic decrease in glutamate-mediated excitatory transmission [18]. It is known that ethanol also affects both neurotransmitters in the CNS, with its effects being complex and dependent on the type of exposure. Acute doses of alcohol increase presynaptic GABA release, stimulate the action of GABA_A_ receptors, and have an inhibitory effect on NMDA receptors. In contrast, chronic exposure to ethanol causes a decrease in the presynaptic release of GABA and an increase in the presynaptic release of glutamate [19]. Furthermore, chronic intermittent ethanol administration reduced the level of 3a-hydroxy-5a-pregnan-20-one (allopregnanolone) in the hippocampus. Allopregnanolone is a positive allosteric modulator of GABA_A_ receptors. Rats with this neurosteroid dysfunction showed impairment of hippocampus-dependent memory function [20]. Another study indicated a significant connection between allopregnanolone hippocampal levels and seizure occurrence in epileptic rats treated with levetiracetam [21].

Moreover, ethanol administration is associated with increased oxidative stress through various mechanisms, including the induction of oxidative damage and lipid peroxidation. Research has demonstrated that alcohol can reduce the overall antioxidant activity in the serum of individuals with alcohol dependence [22] and impair the functions of critical antioxidant enzymes such as superoxide dismutase (SOD), catalase (CAT), and glutathione peroxidases (GPX) [23]. Ethanol consumption leads to prolonged oxidative stress in the brain, which is associated with neuroinflammation and neurodegeneration [24]. In addition, several essential elements (including Cu, Zn, and Fe) are involved in oxidative stress processes and neurodegeneration. Chronic consumption of alcohol may cause absorption disorders and deficiencies in trace elements like Zn and Mg [25]. It has been demonstrated in many preclinical models that levetiracetam has neuroprotective activity and decreases oxidative stress. Levetiracetam pretreatment prevented lipid peroxidation and preserved normal CAT activity in mice hippocampus after pilocarpine-induced seizures [26]. Levetiracetam also attenuates kainic acid-induced toxicity [27] and rotenone-induced toxicity [28].

Therefore, the current study’s objective was to examine whether levetiracetam has a protective activity against ethanol-induced memory impairment, alterations in selected neurotransmitter levels, and oxidative stress in rats. The present work determines, for the first time, the effect of levetiracetam and ethanol on GABA and glutamate concentrations in three rat brain regions (cerebellum, hippocampus, and cerebral cortex), as well as the effect of the drug on serum oxidative stress parameters and the concentration of selected essential elements in the rat brain.

## Materials and methods

### Animals

55 male Wistar rats (251–278 g) were purchased from the Mossakowski Institute of Experimental and Clinical Medicine (Warsaw, Poland). The rats were maintained with a 12-h/12 h light/dark cycle (lights on at 7:00 a.m.) in a temperature-controlled room (20–22 °C) with a relative humidity of 55 ± 5%. All animals were allowed to acclimate to the conditions of the animal facility for one week before the experiment started. The animals were housed in standard cages with plastic tunnels and had free access to commercial chow. Experiments were conducted between 8:00 a.m. − 4:00 p.m. Animals were brought to the testing room 30 min before the start of each behavioral test. The study was performed in compliance with European Union Directive 2010/63/EU, ARRIVE guidelines, and Polish governmental regulations regarding experiments on animals (Dz.U.05.33.289). All experimental protocols were approved by the Local Ethics Committee for Experimentation on Animals in Łódź (no. 9) (resolutions no. 66/ŁB/121/2018; 9/ŁB231/2022).

### Drugs

In the initial phase, 24 animals were divided into three groups (*n* = 8 in each group): levetiracetam and ethanol group (LEV + ET), ethanol group (ET), and control group (C) and participated in the Morris Water Maze test (MWM). Rats were assigned to the treatment groups based on their body mass. Before assigning them to groups, all animals were weighed, ranked from lightest to heaviest, and then assigned to groups in an equal manner. Subsequently, the other 30 animals performed the Novel Object Recognition test (NOR) and were sacrificed for further biochemical analysis. They were divided into three groups (*n* = 8)–levetiracetam (LEV), ET and LEV + ET groups, and control group (C) (*n* = 6). Rats were also assigned to the treatment groups according to body mass. The results of the LEV group in the NOR test have been previously published [29].

Levetiracetam was administered as a ready-made solution (Trund^®^, 10 mg/ml solution; Glenmark Pharmaceuticals, Lot 77065 and 90337) once a day at a dose of 300 mg/kg b.w. directly into the stomach via an oral gavage. The dose was selected based on a review of the available literature [30]. The forced ethanol intake model by Majchrowicz [31] with Szmigielski’s amendment [32] was used to eliminate the risk that the potential observed changes will be caused by variations in alcohol intake. Appropriate alcohol concentrations were prepared from 95% food-grade ethanol by diluting it with water. The LEV + ET and ET groups were given 20% ethanol twice daily in two doses: 1.5 g/kg b.w. (0.75 ml/100 g; in the morning) and 3.5 g/kg b.w. (1.75 ml/100 g; in the afternoon) via an oral gavage. In addition, animals had free access to 5% ethanol between 4.00 p.m. and 8.00 a.m., while water was only available for free drinking between 8 a.m. − 4 p.m. The C and LEV groups had free access to water 24 h a day. Control rats received 1% methylcellulose solution in the same amount as levetiracetam (0.2 ml/100 g). Detailed time administration is shown in Table 1. In the MWM groups, administration of the drug and ethanol was started after an MWM training phase.

**Table 1:** Dosage and time administration of levetiracetam, methylcellulose, ethanol and water

MWM				
Groups	Levetiracetam (300 mg/kg b.w. po) once a day	1% methylcellulose once a day	20% ethanol (5 g/kg po in two doses) + 5% ethanol (afternoon and night access)	Water
C (*n* = 8)	-	three weeks before MWM and during two weeks of MWM	-	access 24 h/day
ET (*n* = 8)	-	-	three weeks before MWM and during first week of MWM	access between 8 a.m. − 4 p.m.
LEV + ET (*n* = 8)	three weeks before MWM and during two weeks of MWM	-	three weeks before MWM and during first week of MWM	access between 8 a.m. − 4 p.m
**NOR + biochemical analysis**				
C (*n* = 6)	-	three weeks before NOR and during NOR	-	access 24 h/day
ET (*n* = 8)	-	-	three weeks before NOR and during NOR	access between 8 a.m. − 4 p.m.
LEV (*n* = 8)	three weeks before NOR and during NOR	-	-	access 24 h/day
LEV + ET (*n* = 8)	three weeks before NOR and during NOR	-	three weeks before NOR and during NOR	access between 8 a.m. − 4 p.m.

*Abbreviations* MWM – Morris water maze, NOR – novel object recognition, C – control group, ET – ethanol group, LEV – levetiracetam group, LEV + ET – levetiracetam and ethanol group, *po* – per os

### Behavioral testing

#### The Morris Water Maze (MWM)

The MWM is used to assess spatial memory and learning in rodents. The test protocol has been thoroughly described in our previous study [29]. Briefly, the MWM was performed in a circular pool (180 cm diameter, 50 cm high) filled with 22 ± 2 ℃ water. The pool was virtually divided into four quadrants, and a submerged platform was placed in one of them. During the three-day training session, rats were familiarized with the experimental scheme. The platform remained in the same quadrant every day. The animal performed four trials a day, with a 60-second interval between them. Each trial started in a different quadrant and lasted a maximum of 60 s. When the rat found the platform, it stayed on it for 15 s to remember its location. If the animal did not find the platform, the experimenter placed it there and allowed the rat to stay on the platform for 15 s. On the fourth day, the retention test was performed following a similar procedure without the hidden platform (one trial). When the rat could not locate the platform in the previous place, it searched for it along the entire pool. The training sessions took place before the administration of levetiracetam and ethanol began. After three weeks of co-administering the drug and alcohol, the tests were repeated with the platform in a different quadrant. The MWM was also conducted 24 h after the discontinuation of ethanol administration. The escape latency, swimming distance, and percentage of time spent in the target zone were measured. Rat position, swimming, and entrance to the platform were recorded and detected using ANY-maze software (ANY-maze, USA). The inability to find the platform faster on subsequent training days of the experiment was a factor excluding the animal from the experiment. No animal was excluded from the experiment.

#### The Novel object recognition test (NOR)

The NOR allows the assessment of recognition memory in rodents and was performed as described previously [29]. The experimental equipment for this test is an empty plastic box. On the first day (habituation phase), rats were put in a box to get accustomed for five minutes. On the next day, the familiarization phase was conducted, where the animal was placed into the same box with two identical objects (A1 and A2) to explore them for three minutes. After five minutes from the exposition to identical objects, one of the familiar objects was replaced with a novel one (B), and the rat was allowed to explore for another three minutes. After 24 h, object B was replaced with a new, previously unused object C, and the animal was allowed to explore for three minutes. Object exploration was defined as directing the snout at the object at no more than one cm distance, sniffing or touching the object with the nose. Sample and test sessions were recorded and analyzed by two investigators blind to treatment conditions with the aid of self-developed software. The discrimination index (DI) was calculated using the following equation: (novel object exploration (s)/both objects exploration (s)) x 100%.

### Biochemical analysis

#### Blood ethanol level determination

On the 28th day of the study, 30 min after the administration of the last morning dose of ethanol, the rats were qualified for euthanasia by decapitation. The whole blood samples were collected for assays. The concentration of ethanol in blood was measured using an enzymatic method and expressed as g/l (‰).

### GABA and glutamate levels determination in selected rats’ brain structures

GABA and glutamate concentrations were evaluated in three rat brain regions (cerebellum, hippocampus, and cerebral cortex) by the UPLC-FLD method, as previously described [33]. The concentrations of determined neurotransmitter values were expressed as µg/g wet tissue.

### Assay of serum oxidative stress markers

The rat serum was obtained after centrifugation of whole blood and used for determination of: TBARS (Thiobarbituric acid reactive substances, TBARS Assay Kit, Cayman Chemical, no. 700870), SOD (Superoxide Dismutase, Superoxide Dismutase Assay Kit, Cayman Chemical, no. 706002) and CAT (catalase) according to the respective manufacturer’s instructions (Catalase Assay Kit, Cayman Chemical, no. 707002).

### Assay of selected essential elements in the brain

The brain essential elements (Cu, Mn, Zn, Fe, Mg) concentrations were performed by flame atomic spectrometry (F-AAS) and flameless atomic spectrometry (GF-AAS). Instrumental conditions of F-AAS and GF-AAS were investigated for each inorganic element. The brain samples were digested with ultrapure HNO_3_ and H_2_O_2_, using a microwave digestion system (MARSXpress, CEM Corporation, USA) [34]. Brain Cu and Mn concentrations were measured using a Hitachi Z-8270 GFAAS (Hitachi, Ltd., Tokyo, Japan) with a Zeeman-type background correction, an autosampler, and a pyro-coated tube. The levels of brain Zn, Fe, and Mg were determined using flame atomic absorption spectrometry on F-AAS (Avanta GM; GBC Scientific Equipment Pty Ltd., Melbourne, Australia). For each series of analyses, internal quality controls were used (Bovine liver 1577b (National Institute of Standards and Technology)). For 96% of the determinations, the repeatability error did not exceed 10%. The detection limits (LOD) were as follows: Cu (0.04 µg/g wet tissue), Mn (0.06 µg/g wet tissue), Zn (0.01 µg/g wet tissue), Fe (0.03 µg/g wet tissue), and Mg (0.3 µg/g wet tissue). The concentrations of essential elements in the brain were expressed as µg/g wet tissue.

### Statistical analysis

All data were processed by Statistica 13.3 software. The data were tested for normality and the homogeneity of variances using the Kolmogorov-Smirnov test with Lilliefors correction and Levene’s test, respectively. A one-way analysis of variance (ANOVA), followed by Tukey’s multiple comparisons test, was used to compare the means in the NOR test and ethanol blood level. Due to the lack of normal distribution or homogeneity of variance, some data were evaluated by the non-parametric tests: the Kruskal-Wallis test to compare groups on the particular test day and the Friedman rank sums test to compare different test days within each group. Both non-parametric tests were used to analyze results from MWM. The Kruskal-Wallis test was used to compare the concentrations of Cu and Mg between groups. For all other results, a two-way analysis of variance (ANOVA) was used, followed by Sidak’s multiple comparisons test. Differences were considered significant at *p* < 0.05. The data reported in this paper were presented as mean ± standard error of the mean (SEM) (parametric tests) or median (horizontal bar), first and third quartiles (vertical column), and minimum and maximum (vertical line) (non-parametric tests). Outlier values are represented with circles.

## Results

### Morris water maze test

#### Initial training before levetiracetam and ethanol administration

All rats performed training tasks equally well and showed no signs of impairment of motor and visual abilities. The time needed to find the platform and swimming distance gradually shortened during the following days in all groups. The time spent in the target zone with the platform increased during training (data not shown).

#### The effect of co‑administration of levetiracetam and ethanol on the spatial memory in rats

**Fig. 1:**
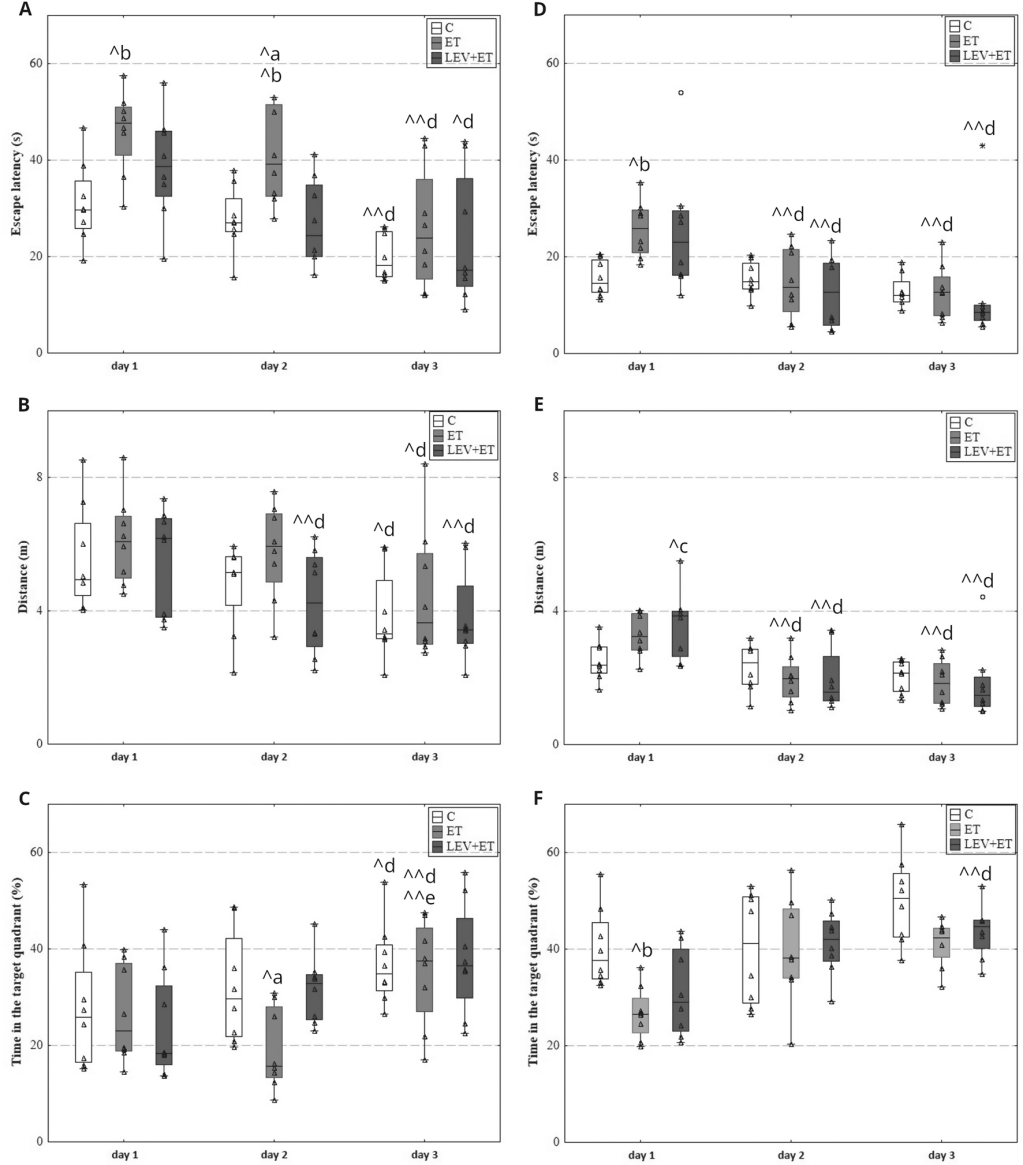
Effect of three weeks of levetiracetam (300 mg/kg b.w. *po*) and ethanol administration in the Morris Water Maze (MWM) on time needed to localize the platform (**A**), the distance traveled–(**B**), the time spent in the zone with the platform (**C**), and four weeks of levetiracetam administration after 24 h from discontinuation of ethanol administration in MWM on time needed to localize the platform (**D**), the distance traveled–(**E**), the time spent in the zone with the platform (**F**); ET–ethanol group (*n* = 8), C–control group (*n* = 8), LEV + ET–levetiracetam and ethanol group (*n* = 8);. ^a^ Statistically significant difference between ET and C groups on a particular test day; the Kruskal-Wallis test. ^b^ Statistically significant difference between ET and LEV + ET groups on a particular test day; Kruskal-Wallis test. ^c^ Statistically significant difference between C and LEV + ET groups on a particular test day; Kruskal-Wallis test. ^d^ Statistically significant difference between a particular test day and test day 1; the Friedman’s test. ^e^ Statistically significant difference between a particular test day and test day 2; the Friedman’s test. ^*p* < 0.05, ^^*p* < 0.01; the data are presented as box plots, with the horizontal line indicating the median, and vertical boxes and whiskers depicting the percentile range; outlier values are represented with circles

Kruskal-Wallis test revealed that three weeks of ethanol administration disrupted spatial memory in rats, and levetiracetam may improve it. On the second test day, levetiracetam significantly decreased the latency to the hidden platform (H(2, *N* = 24) = 7.94, *p* = 0.019) (Fig. 1A) and increased the percentage of time spent in the current goal quadrant compared to the ethanol group (H(2, *N* = 24) = 7.04, *p* = 0.030) (Fig. 1C). A significant ethanol-induced increase in escape latency was also observed on days 1 and 2 compared to the control group (respectively H(2, *N* = 24) = 7.45, *p* = 0.024; H(2, *N* = 24) = 7.94, *p* = 0.019) (Fig. 1A). However, the escape latency and distance traveled of all groups significantly decreased on day 3 compared to initial values (escape latency: ET–Chi sq = 9.75, *N* = 8, *p* = 0.008; C–Chi sq = 9.75, *N* = 8, *p* = 0.008; LEV + ET–Chi sq = 7.75, *N* = 8, *p* = 0.021; distance: ET–Chi sq = 7.00, *N* = 8, *p* = 0.030; C–Chi sq = 7.00, *N* = 8, *p* = 0.030; LEV + ET–Chi sq = 9.25, *N* = 8, *p* = 0.009) (Fig. 1A, B). In the LEV + ET group, this significant decrease in distance was also noted on day 2 compared to the initial values (Chi sq = 9.25, *N* = 8, *p* = 0.009) (Fig. 1B). On day 3, rats from the C and ET groups spent significantly more time in the target quadrant compared to the initial values (respectively Chi sq = 6.25, *N* = 8, *p* = 0.044; Chi sq = 10.75, *N* = 8, *p* = 0.005) and compared to day 2 in the ET group (Chi sq = 6.25, *N* = 8, *p* = 0.044) (Fig. 1C).

#### The effect of levetiracetam on the spatial memory in rats after 24 h from discontinuation of ethanol administration

Kruskal-Wallis test revealed that memory impairment maintained in rats previously receiving ethanol and levetiracetam had no beneficial effect. On day 1, the animals from the ET group needed more time to find the hidden platform (H(2, *N* = 24) = 7.98, *p* = 0.019) (Fig. 1D) and spent less time in the target quadrant compared to the C group (H(2, *N* = 24) = 8.80, *p* = 0.012) (Fig. 1F). Additionally, the LEV + ET group swam longer distance compared to the C group (H(2, *N* = 24) = 6.52, *p* = 0.039) (Fig. 1E). However, the disturbances observed in these animals had a temporary effect as significant decreases were noticed in escape latency and distance traveled on days 2 and 3 compared to the initial values (escape latency: ET–Chi sq = 12.00, *N* = 8, *p* = 0.002; LEV + ET–Chi sq = 12.25, *N* = 8, *p* = 0.002; distance: ET–Chi sq = 9.25, *N* = 8, *p* = 0.009; LEV + ET–Chi sq = 13.00, *N* = 8, *p* = 0.002) (Fig. 1D, E). On the last day of MWM, a significant increase in the percentage of time in the current goal quadrant was also noted in the LEV + ET group (Chi sq = 9.75, *N* = 8, *p* = 0.008) (Fig. 1F).

### Novel object recognition

#### The effect of co-administration of levetiracetam and ethanol on the short- and long-term memory in rats

**Fig. 2:**
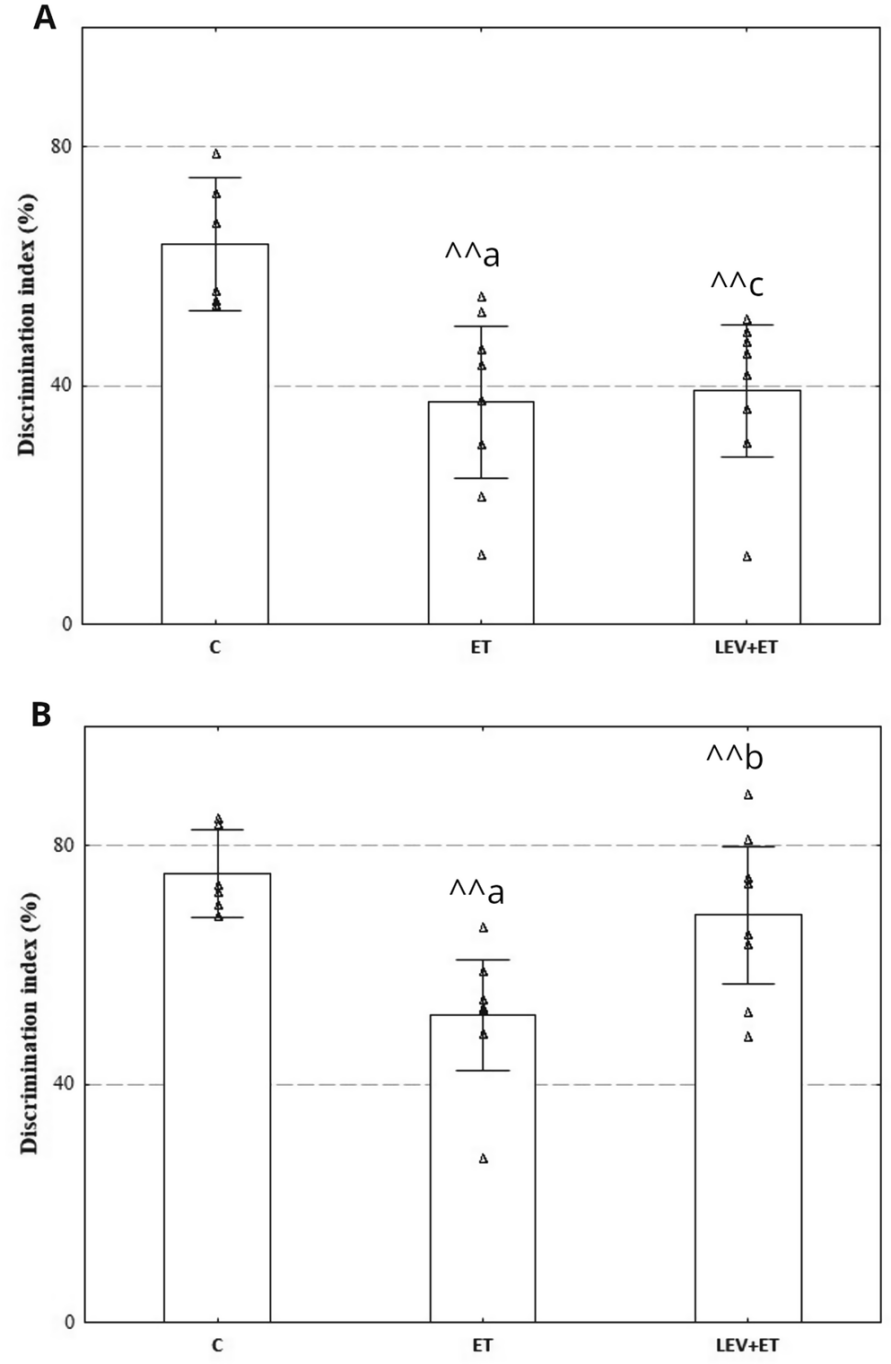
Effect of three weeks of levetiracetam (300 mg/kg b.w. *po*) and ethanol administration on short-term (**A**) and long-term recognition memory (**B**); ET–ethanol group (*n* = 8), C–control group (*n* = 6), LEV + ET–levetiracetam and ethanol group (*n* = 8);. ^a^ Statistically significant difference between ET and C groups; one-way ANOVA, Tukey’s post hoc test. ^b^ Statistically significant difference between ET and LEV + ET; one-way ANOVA, Tukey’s post hoc test. ^c^ Statistically significant difference between C and LEV + ET groups; one-way ANOVA, Tukey’s post hoc test. ^^*p* < 0.01; the data are presented as mean ± standard error of the mean (SEM)

One-way ANOVA revealed that ethanol and levetiracetam affected recognition memory. Ethanol administered for three weeks impaired short- and long-term memory in rats and significantly decreased the discrimination index compared to the C group (respectively F_2,19_=7.99, *p* = 0.003; F_2,19_=8.26, *p* = 0.003) (Fig. 2A, B). Levetiracetam co-administered with ethanol significantly improved long-term memory compared to the ET group (F_2,19_=7.99, *p* = 0.003) but did not affect short-term recognition memory. The discrimination index in the short-term memory test was significantly lower in the LEV + ET group compared to the C group (F_2,19_=7.99, *p* = 0.003).

### Blood ethanol level

There was no difference observed in blood ethanol levels between rats receiving ethanol and levetiracetam and those receiving ethanol alone (Supplementary material 1).

#### The effect of levetiracetam and ethanol on GABA and glutamate levels in selected rats’ brain structures

**Fig. 3:**
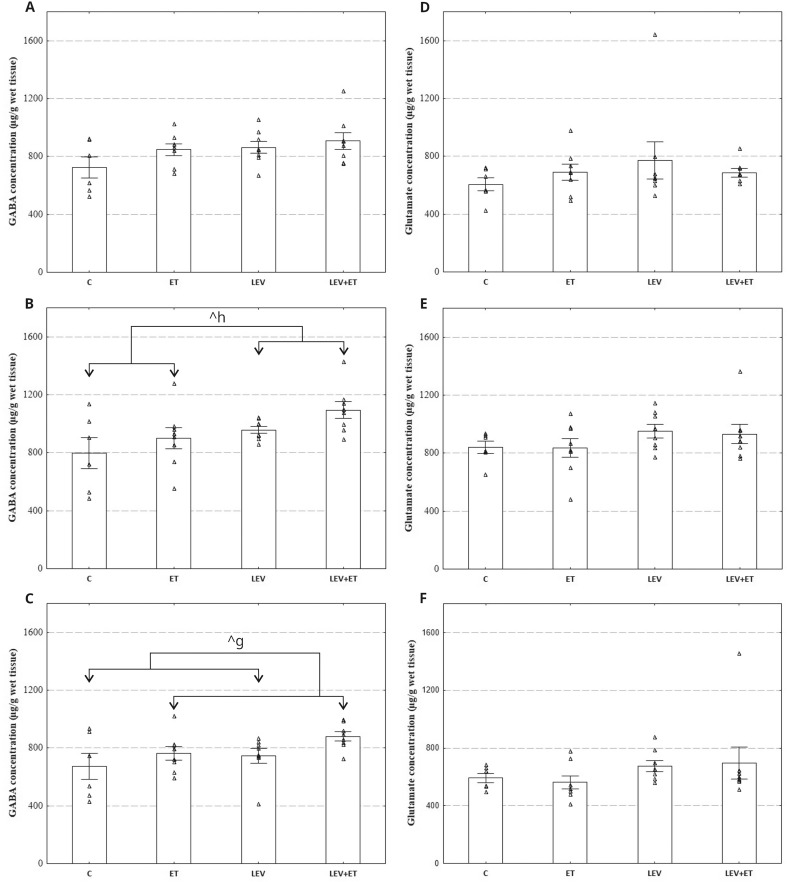
Effect of four weeks of levetiracetam (300 mg/kg b.w. *po*) and ethanol administration on GABA (**A**, **B**, **C**) and glutamate levels (**D**, **E**, **F**) in the hippocampus (**A**, **D**), cerebral cortex (**B**, **E**), and cerebellum (**C**, **F**); ET–ethanol group (*n* = 8), C–control group (*n* = 6), LEV–levetiracetam group (*n* = 8), LEV + ET–levetiracetam and ethanol group (*n* = 8);. ^g^ Statistically significant difference between all rats receiving ethanol and all non-alcoholic rats; two-way ANOVA. ^h^ Statistically significant difference between all rats receiving levetiracetam and all non-drug rats; two-way ANOVA. ^*p* < 0.05; the data are presented as mean ± standard error of the mean (SEM)

A two-way ANOVA revealed that levetiracetam and ethanol did not affect glutamate concentrations in the hippocampus (respectively F_1,26_=1.063, *p* = 0.3121; F_1,26_=0.0001, *p* = 0.9902), cerebral cortex (respectively F_1,26_=3.108, *p* = 0.0896; F_1,26_=0.04523, *p* = 0.8332), and cerebellum (respectively F_1,26_=2.472, *p* = 0.1280; F_1,26_=0.00514, *p* = 0.9434) (Fig. 3D-F). Glutamate concentration was also not affected by levetiracetam x ethanol interaction in the hippocampus (F_1,26_=1.176, *p* = 0.2882), cerebral cortex (F_1,26_=0.01865, *p* = 0.8924) and cerebellum (F_1,26_=0.1528, *p* = 0.6991). A two-way ANOVA revealed that GABA concentration was also not influenced by levetiracetam, ethanol, and levetiracetam x ethanol interaction in the hippocampus (respectively F_1,26_=3.601, *p* = 0.0689; F_1,26_=2.552, *p* = 0.1222; F_1,26_=0.5406, *p* = 0.4688) (Fig. 3A). In the cerebral cortex, GABA concentration was significantly influenced by levetiracetam (F_1,26_=6.831, *p* = 0.0147) (Fig. 3B). However, it was not affected by ethanol (F_1,26_=3.154, *p* = 0.0875) nor levetiracetam x ethanol interaction occurred (F_1,26_=0.06281, *p* = 0.8041). Moreover, GABA concentration in the cerebellum was affected by ethanol (F_1,26_=4.228, *p* = 0.0499), but levetiracetam (F_1,26_=3.028, *p* = 0.0937) nor levetiracetam x ethanol interaction were significant (F_1,26_=0.1598, *p* = 0.6926) (Fig. 3C).

#### The effect of levetiracetam and ethanol on serum oxidative stress parameters

**Fig. 4:**
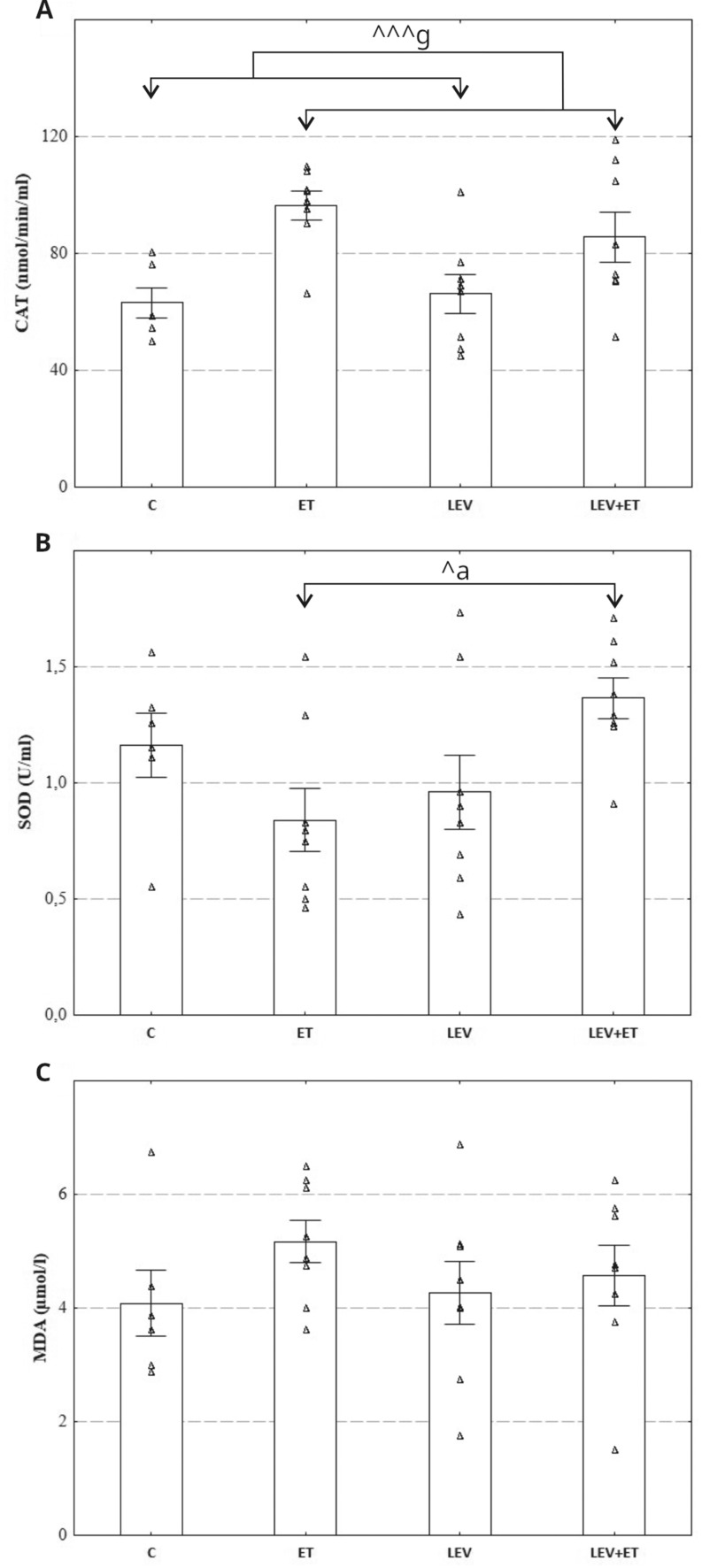
Effect of four weeks of levetiracetam (300 mg/kg b.w. *po*) and ethanol administration on catalase (**A**), superoxide dismutase (**B**), and malondialdehyde (**C**) in the serum of rat; ET–ethanol group (*n* = 8), C–control group (*n* = 6), LEV–levetiracetam group (*n* = 8), LEV + ET–levetiracetam and ethanol group (*n* = 8);. ^a^ Statistically significant difference between ET and LEV + ET groups; two-way ANOVA, Sidak’s multiple comparisons test. ^g^ Statistically significant difference between all rats receiving ethanol and all rats receiving water; two-way ANOVA. ^*p* < 0.05, ^^^ *p* < 0.001; the data are presented as mean ± standard error of the mean (SEM)

A two-way ANOVA showed that ethanol had a significant influence on CAT activity (F_1,26_=15.79, *p* = 0.0005), whereas levetiracetam (F_1,26_=0.3401, *p* = 0.5648) and the levetiracetam x ethanol interaction (F_1,26_=1.106, *p* = 0.3026) were not significant (Fig. 4A, B). Additionally, SOD activity was affected by levetiracetam x ethanol interaction (F_1,26_= 7.213, *p* = 0.0124) but levetiracetam (F_1,26_=1.439, *p* = 0.2411) or ethanol (F_1,26_=0.09693, *p* = 0.7580) exerted no significant effect. Sidak’s multiple comparisons test showed that co-administration of levetiracetam with ethanol significantly increased SOD activity compared to the ethanol group. A two-way ANOVA revealed that MDA level was not affected by levetiracetam (F_1,26_=0.1656, *p* = 0.6873), ethanol (F_1,26_=1.860, *p* = 0.1843) or levetiracetam x ethanol interaction (F_1,26_=0.5736, *p* = 0.4556) (Fig. 4C).

#### The effect of levetiracetam and ethanol on selected essential element concentrations in the brain

**Fig. 5:**
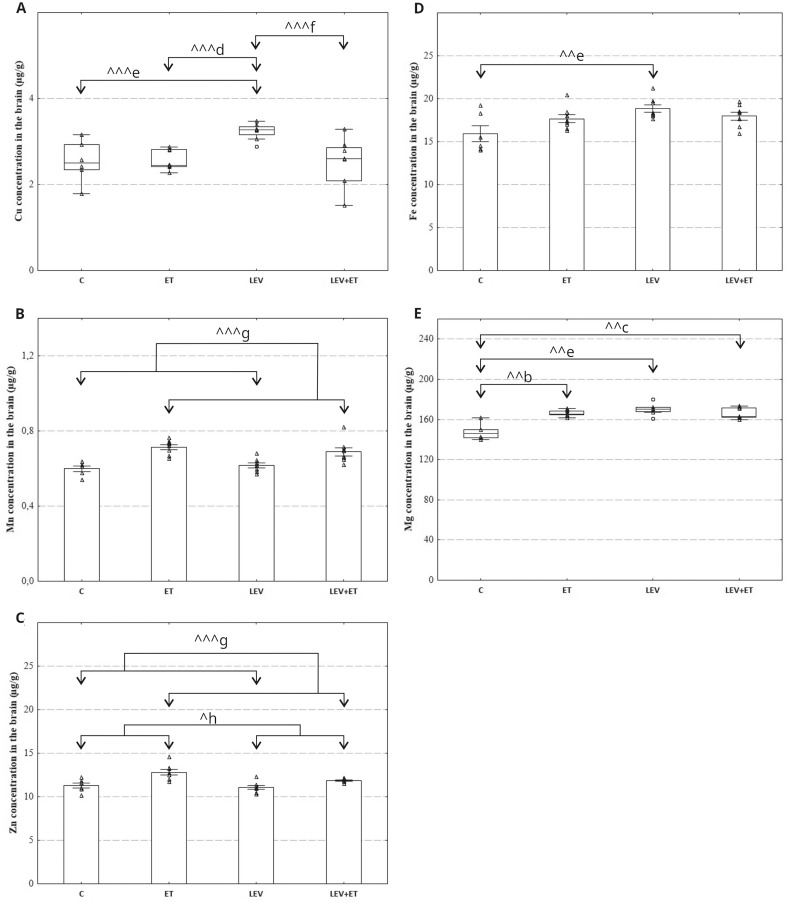
Effect of four weeks of levetiracetam (300 mg/kg b.w. *po*) and ethanol administration on copper (**A**), manganese (**B**), zinc (**C**), iron (**D**) and magnesium concentration (**E**) in the brain of rat; ET–ethanol group (*n* = 8), C–control group (*n* = 6), LEV–levetiracetam group (*n* = 8), LEV + ET–levetiracetam and ethanol group (*n* = 8);. ^b^ Statistically significant difference between ET and C groups; Kruskal-Wallis test. ^c^ Statistically significant difference between C and LEV + ET groups; Kruskal-Wallis test. ^d^ Statistically significant difference between ET and LEV groups; Kruskal-Wallis test. ^e^ Statistically significant difference between C and LEV groups; two-way ANOVA, Sidak’s multiple comparisons test (**D**) or Kruskal-Wallis test (**A**, **E**);. ^f^ Statistically significant difference between LEV + ET and LEV groups; Kruskal-Wallis test. ^g^ Statistically significant difference between all rats receiving ethanol and all non-alcoholic rats; two-way ANOVA. ^h^ Statistically significant difference between all rats receiving levetiracetam and all non-drug rats; two-way ANOVA. ^*p* < 0.05, ^^*p* < 0.01, ^^^*p* < 0.001; the data are presented as box plots, with the horizontal line indicating the median, and vertical boxes and whiskers depicting the percentile range (**A**, **E**) or as mean ± standard error of the mean (SEM) (**B**, **C**, **D**)

The statistical analysis revealed that both levetiracetam and ethanol had an impact on all studied essential element concentrations. A two-way ANOVA showed that the concentration of Mn was significantly affected by ethanol (F_1,26_=33.08, *p* < 0.0001), while the effect of levetiracetam (F_1,26_=0.03747, *p* = 0.8480) and levetiracetam x ethanol interaction were insignificant (F_1,26_=1.720, *p* = 0.2012) (Fig. 5B). Additionally, the concentration of Zn was significantly influenced by both ethanol (F_1,26_=23.44, *p* < 0.0001) and levetiracetam (F_1,26_=6.149, *p* = 0.0199) but levetiracetam x ethanol interaction was not significant (F_1,26_ = 2.414, *p* = 0.1323) (Fig. 5C). Furthermore, the two-way ANOVA also revealed that levetiracetam (F_1,26_=8.591, *p* = 0.0070), as well as levetiracetam x ethanol interaction (F_1,26_=5.642, *p* = 0.0252), were significant for Fe concentration (Fig. 5D). However, there was no effect of ethanol (F_1,26_= 0.5610, *p* = 0.4606). Sidak’s multiple comparisons test indicated that levetiracetam significantly increased Fe concentration compared to controls. Additionally, levetiracetam administered alone increased the concentration of Cu in the examined brain samples compared to all studied groups (H(3, *N* = 30) = 13.73, *p* = 0.003). Similarly to Cu concentration, a significant increase in Mg concentration was noted in the brains of animals receiving levetiracetam, and this effect differed significantly from the control group (H(3, *N* = 30) = 15.03, *p* = 0.002). Moreover, animals receiving levetiracetam co-administered with ethanol or ethanol alone also showed increased Mg concentration compared to the control group (H(3, *N* = 30) = 15.03, *p* = 0.002).

## Discussion

The present study shows that levetiracetam has varying effects on ethanol-induced memory impairment in rats. The drug administered with ethanol alleviated spatial memory disorders in the MWM but did not affect them after discontinuation of alcohol administration. Additionally, levetiracetam improved long-term recognition memory but did not affect ethanol-induced short-term memory impairment in the NOR test. These observed post-ethanol disorders do not seem to be linked to changes in neurotransmitter concentrations. In this study, it was found that ethanol only had an impact on GABA concentration in the cerebellum, and levetiracetam had an impact on GABA concentration in the cerebral cortex. However, it was observed that alcohol might induce oxidative stress by increasing CAT activity and decreasing SOD activity. Levetiracetam reversed the adverse effect of alcohol on SOD activity. Furthermore, ethanol disturbed the homeostasis of essential elements such as Mn, Zn, and Mg in the rat brain. The effect of levetiracetam on these essential elements was mixed, as it decreased Zn level while elevated the Cu, Mg and Fe levels in the rat brain which may potentially lead to the induction of oxidative stress.

In the initial part of this study, the MWM test was used to assess spatial memory. Our studies showed that ethanol temporarily disrupted the rats’ performance in the test, indicating an impairment in their spatial memory. Levetiracetam alleviated these effects, particularly on the second day of testing, resulting in improved escape latency and increased time spent in the target zone. However, the drug did not prevent the memory impairment that occurred after discontinuation of alcohol administration. The effects of alcohol were predominantly observed on the first day of testing.

Levetiracetam has been found to attenuate spatial memory deficits associated with diabetes [35] and in the streptozotocin-induced rat model of Alzheimer’s disease [36]. This study appears to be the first to investigate the impact of levetiracetam on spatial memory in rodents receiving ethanol using MWM. The available literature provides only one clinical study evaluating its effect on cognitive function in patients with alcohol dependence. In this study, levetiracetam administered for 14 weeks *po* at a dose of 2000 mg/day did not affect verbal fluency and verbal working memory as assessed by neuropsychological tests. However, impairment of these cognitive functions was observed in patients receiving other antiseizure medications, such as topiramate and zonisamide [2]. Cognitive impairment is a common side effect of anticonvulsants, and therefore, the impact of levetiracetam alone on memory was evaluated in our earlier study. We demonstrated that the drug administered *po* as a single (100 mg/kg or 500 mg/kg) or repeated doses (300 mg/kg) did not alter spatial memory in rats [29]. A similar absence of memory impairment was also observed in normal and amygdala-kindled rats receiving levetiracetam *ip* for seven days at doses of 17, 54, and 170 mg/kg [37]. On the other hand, Sarangi et al. (2016) found that levetiracetam administered for 45 days at a dose of 310 mg/kg *po* impairs spatial memory, which was associated with longer escape latency [30].

Promising findings of MWM prompted us to further explore this area, with a focus on recognition memory in the NOR test as the next step in our experiment. As in the MWM task, ethanol was found to disrupt both short- and long-term memory. Levetiracetam administered for three weeks with ethanol improved only ethanol-induced long-term memory impairment in animals. However, as we already published in our previous study, the drug administered alone also impaired long-term memory [29]. As was mentioned above, there is currently no literature describing the effect of levetiracetam on hippocampal function and memory processes in animals receiving ethanol. Nevertheless, the impact of the drug on cognitive processes was assessed using the NOR in other models. Recent findings indicated a protective effect of levetiracetam against cognitive impairment induced by lipopolysaccharides. The drug (100 or 200 mg/kg) administrated orally for 30 days improved the exploration of novel object [38]. Moreover, Rehman et al. (2022) evaluated the effect of levetiracetam on recognition memory in pentylenetetrazole-kindled rats. Levetiracetam was administered at a dose of 50 mg/kg *ip* during an 11-day PTZ-kindling procedure. It was observed that the levetiracetam-treated animals significantly preferred the novel object compared to the control group [39]. Moreover, administration of levetiracetam to adult rats attenuated adolescent stress-induced impairment of object recognition memory [40] and improved short-term memory in a mouse model of chronic cerebral hypoperfusion [41].

In the present study, ethanol concentration did not significantly differ between groups due to the forced ethanol intake model. This method was chosen to ensure that any behavioral and biochemical changes were not due to variations in ethanol consumption. Our investigation aimed to determine whether the observed ethanol-induced impairment in recognition memory in the NOR task could be related to changes in central neurotransmission. It is known that ethanol exposure alters GABAergic and glutamatergic synaptic transmissions, and this modulatory effect is varied. Acute exposure to ethanol stimulates GABA transmission and inhibits glutamatergic transmission, while chronic exposure produces the opposite effects [19]. Recognition memory depends on the perirhinal cortex and its functional interactions with the hippocampus [42]. Additionally, the medial prefrontal cortex is involved in the recognition memory [43]. In turn, the cerebellum plays a role in motor function, but it also contributes to cognitive functions [44]. In our study, ethanol increased GABA concentration only in the cerebellum, compared to animals receiving only water. Interpreting these results is challenging, but the lack of expressed impact of ethanol might be due to the relatively short-term 28-day study. Therefore, the lack of effects from chronic exposure to ethanol cannot be excluded. It has been demonstrated that ethanol altered GABA_A_ receptor gene expression in the cerebellum [45]. Our results are not in line with those of previous studies that demonstrated ethanol-induced cognitive impairment through alterations of GABA and glutamatergic neurotransmission in the hippocampus and cortex [46–49]. Ethanol-induced glutamate alterations were also observed in the cerebellum [50]. In the present study, levetiracetam also increased GABA level in the cerebral cortex. It has been previously shown that levetiracetam affects GABA transporters [17] and reverses the Zn²⁺-induced suppression of GABA-mediated inhibition in the hippocampus [18].

Ethanol exposure is known to cause oxidative stress by triggering the production of reactive oxygen species and inhibiting the function of antioxidant proteins and enzymes. Studies indicate that alcohol can disrupt the activities of antioxidants such as SOD, CAT, and GPX activities. Peng et al. (2005) demonstrated that SOD and GPX activities were reduced in individuals with alcohol dependence, and the decrease in SOD activity persisted even after 14 days of abstinence. Additionally, the levels of serum MDA, an indicator of oxidative stress, were significantly increased in patients compared with controls, and a decrease was also observed during the abstinence [23]. Similar effects were observed in other clinical studies [51–53]. The impact of ethanol on CAT can vary, as this enzyme is involved in ethanol metabolism. Chronic ethanol consumption increases CAT activity, while shorter consumption decreases its activity [24, 54, 55]. However, some findings indicate that CAT activity might also be reduced in patients with alcohol dependence [23]. Alcohol consumption leads to prolonged oxidative stress throughout the body, including the brain, which is associated with neuroinflammation and neurodegeneration [24]. In the present study, it was observed that serum CAT activity increased while SOD activity decreased. Levetiracetam significantly reversed the ethanol-induced reduction of SOD activity but did not affect CAT activity. Although the MDA level did not significantly differ between groups, it was highest in the group receiving ethanol. This is the first study investigating the impact of the interaction of levetiracetam and ethanol on oxidative stress parameters. Although the antioxidant properties of the drug have been studied in other research, the evidence is inconclusive. Different studies have shown that levetiracetam may either reduce [30] or augment oxidative stress [37]. Some studies also demonstrated that the drug has a neuroprotective effect in the brain [28] and attenuated oxidative stress in the hippocampus [26]. Our findings showed a positive tendency regarding the decrease of oxidative stress by levetiracetam, but further research is required to assess this impact, especially in the brain.

The concentration of essential elements was also evaluated in our study. It is known that alterations in their concentration occur in individuals with alcohol dependence. Alcohol can disrupt the homeostasis of essential elements. Ion concentration is linked to oxidative stress, with copper serving as a cofactor of antioxidant enzymes, iron being present in the active sites of enzymes responsible for oxidation and reduction reactions, and manganese being part of SOD but also acting as a neurotoxin at higher concentrations. Exceeding optimal ion concentrations may induce various pathological processes, including lipid peroxidation, leading to damage of cell membranes and disturbances of cell integrity [25, 56]. In this study, changes in essential element homeostasis in ethanol-treated rats were observed, with significant increases in the brain levels of Mn, Zn, and Mg. Results from another study also showed an increase in Mn concentration in mice. The authors suggest that increased brain Mn concentration along with upregulation of iron transporters may pose a higher risk of Mn neurotoxicity in alcohol-drinking patients. While increased Fe content was observed in the brains of rats receiving ethanol, it was only significant in the highest dose (10% v/v) [57]. These results differed from ours as no significant changes were noted in rats receiving ethanol in the present study. The available literature on the impact of ethanol on these ions is limited and often inconsistent. Although there were no abnormalities of zinc metabolism in the animals, clinical studies have reported decreases in zinc [58, 59]. Additionally, Mg concentration was decreased after chronic ethanol exposure in rats [59]. When levetiracetam was co-administered with ethanol, it did not affect altered Zn, Mg and Mn concentrations. Furthermore, levetiracetam administered alone increased Cu, Mg, and Fe concentrations while all animals receiving levetiracetam had lower Zn concentrations in the brains. There is no available literature regarding disturbances in element homeostasis after the administration of levetiracetam. The impact of the drug on Fe, Mg and Cu concentrations is worth emphasizing, as high levels of these ions are related to the induction of inflammatory and oxidative stress [60]. These negative observations may be linked to the long-memory impairment observed in levetiracetam-treated rats in the NOR [29].

In summary, our findings suggest that levetiracetam has different effects on memory. When administered with ethanol, the drug improved spatial memory, but not after discontinuation of alcohol administration. Levetiracetam improved long-term recognition memory impairment but did not affect short-term recognition memory in rats receiving ethanol. The negative impact of ethanol was not linked to changes in GABA or glutamate concentrations in the brain, but it may be associated with oxidative stress. Ethanol affected some serum oxidative stress parameters, and levetiracetam reversed the ethanol-induced changes in SOD activity. Ethanol also increased levels of Mn, Zn, and Mg in the brain. Additionally, the drug alone increased Mg, Fe, and Cu concentrations in the brain. All animals receiving levetiracetam also had significantly lower concentrations of Zn. The changes observed in both the brain and serum after administration of the drug, either alone or with alcohol, indicate the need for further research. These findings may have important clinical implications for patients treated with levetiracetam in the future.

## Electronic supplementary material

Below is the link to the electronic supplementary material.

Supplementary Material 1

## Data Availability

The data used in this study are available from the corresponding author upon reasonable request.

## Abbreviations

AMPA: α–amino–3–hydroxy–5–methyl–4–isoxazolepropionic acid ()
ANOVA: Analysis of Variance
b. w.: Body weight
C: Control Group
CAT: Catalase
CNS: Central Nervous System
Cu: Copper
DI: Discrimination Index
ET: Ethanol Group
F-AAS: Flame Atomic Spectrometry
Fe: Iron
GABA: γ–Aminobutyric Acid
GF-AAS: Flameless Atomic Spectrometry
GPX: Glutathione Peroxidases
ip: Intraperitoneal
LEV: Lewetiracetam Group
LEV + ET: Group Receiving Levetiracetam and Ethanol
LOD: Limit Of Detection
MDA: Malondialdehyde
Mg: Magnesium
Mn: Manganese
MWM: Morris Water Maze
NMDA: N–methyl–D–aspartate
NOR: Novel Object Recognition
pharmaco: EEG–pharmaco–electroencephalography
po: Per os
TBARS: Thiobarbituric Acid Reactive Substances
SEM: Standard Error of the Mean
SOD: Superoxide Dismutase
SV2A: Synaptic Vesicle 2 A Protein
UPLC: FLD–ultra–performance liquid chromatography with fluorescence detector method
Zn: Zinc
