# Supplementary material for: Impact of levetiracetam and ethanol on memory, selected neurotransmitter levels, oxidative stress parameters, and essential elements in rats
Source: Pharmacol Rep. 2024 Oct 1;76(6):1363–76. doi: 10.1007/s43440-024-00659-5 (PMC11582331; doi:10.1007/s43440-024-00659-5)
Supplement: Supplementary file 1 — Supplementary Material 1 [file 43440_2024_659_MOESM1_ESM.pdf]

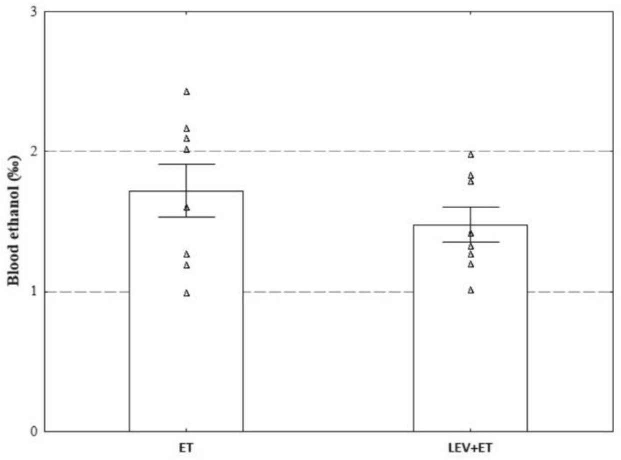

Blood ethanol level; ET – ethanol group (n=8), LEV+ET – levetiracetam and ethanol group (n=8), one-way ANOVA, the data are presented as mean ± standard error of the mean (SEM)
